# Supplementary material for: Contribution of Sorting Nexin 3 in the Cytomegalovirus Assembly
Source: Biomedicines. 2025 Apr 11;13(4):936. doi: 10.3390/biomedicines13040936 (PMC12024572; doi:10.3390/biomedicines13040936)
Supplement: Supplementary file 1 [file biomedicines-13-00936-s001.zip › biomedicines-3521701-supplementary.pdf]

Supplementary material

Supplementary figures

Figure S1

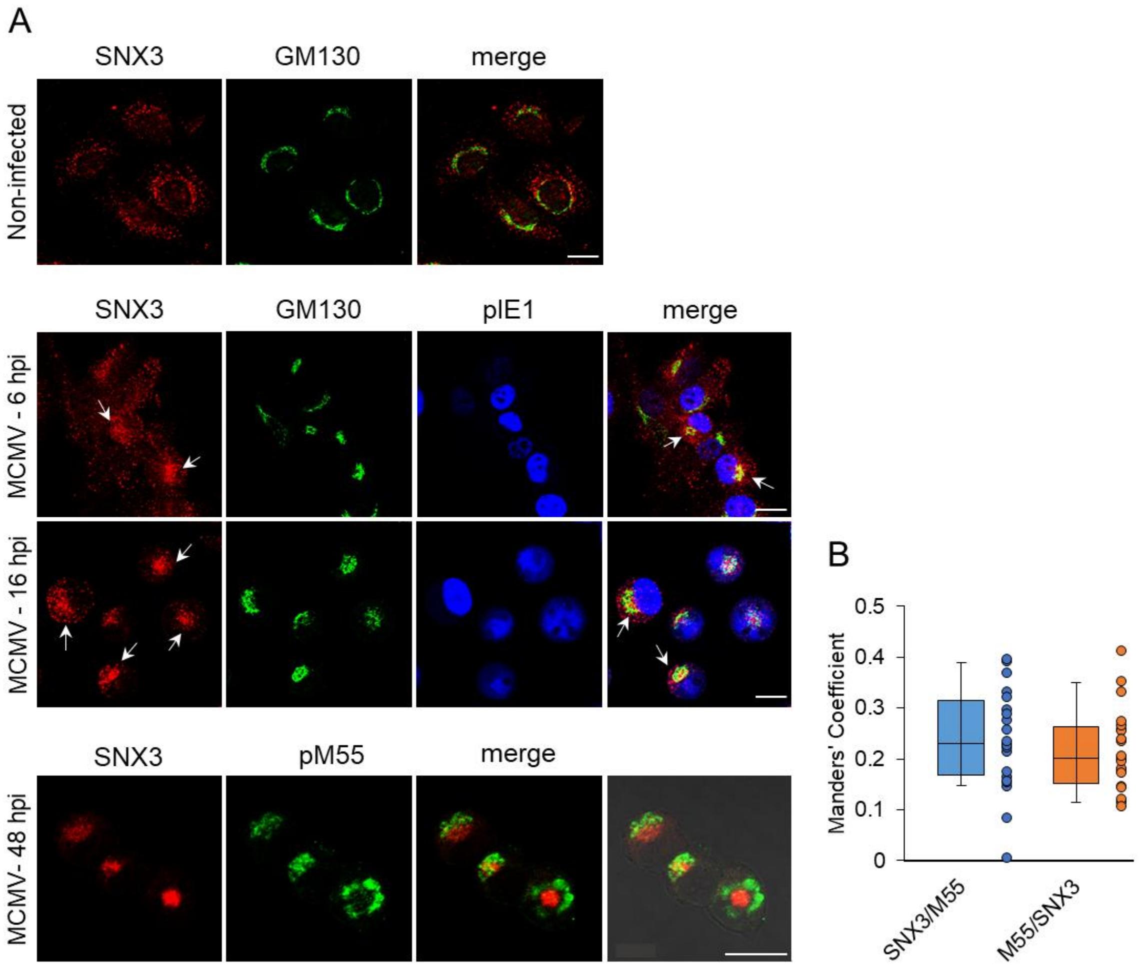

Figure S1. Perinuclear accumulation of SNX3 in the pre-AC and AC of MCMV-infected Balb3T3 cells (*related to Figure 1A*). (**A**) Immunofluorescence analysis of SNX3, GM130, and pM55 in the E (6 and 16 hpi) and L (48 hpi) phase of infection. Balb3T3 cells were infected with Δm138-MCMV (MOI 10) or left non-infected, fixed at 6, 16, and 48 hpi, permeabilized, and stained with Abs against SNX3 (red) in combination with Abs against GM130 or pM55 (green) and pIE1 to control infection (blue). Shown are confocal images through the focal plane of a representative experiment. The arrows indicate perinuclear accumulation in the pre-AC. Bars, 20 µm. (**B**) Colocalization analysis is shown as percentage overlap (Manders' coefficients) of SNX3 with pM55 (M1) and pM55 with SNX3 (M2) per cell. The measurement was performed over the entire Z-stack with the Otsu-algorithm threshold.

Figure S2

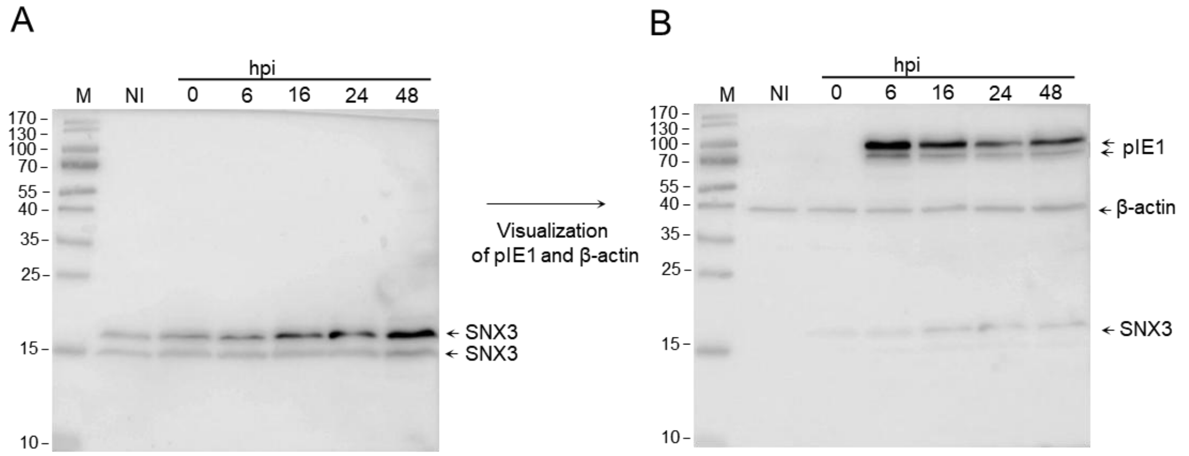

Figure S2. Unprocessed Western blots of SNX3 expression during the MCMV replication cycle (*related to Figure 1C*). Overlaid original raw blots and unprocessed ECL images of SNX3 (**A**), pIE1, and β-actin (**B**), which were used as representative Western blots in Figure 1C of the manuscript. The western blotting membrane was first stained with Ab against SNX3. After visualization of SNX3 with secondary Ab, the membrane was washed and simultaneously stained with Abs against pIE1 and β-actin. The SNX3 protein is shown in the 18 kDa and 15 kDa forms. The molecular weight marker used in these experiments is PageRuler Prestained Protein Ladder (Cat. No. 26616; Thermo Fisher Scientific, Waltham, MA, SAD). M, marker line; NI, non-infected; 0-48, preparation time of infected cell samples.

NIH3T3 cells were plated in 12-well plates ($6x10^4$ cells/well) and infected the next day with Δm138-MCMV (virus ΔMC95.15) at an MOI of 10. Samples were collected immediately (0 hpi) and at different time points after infection (6-48 hpi). Non-infected (NI) cells served as a control. Cells were detached with a short trypsin/EDTA treatment (120 µl/well), trypsin was neutralized with 1.2 ml tissue culture medium containing 10% FCS, pelleted at 2,200 rpm for 2.5 min, and washed twice with PBS. The pellet was lysed on ice in 20 µL of lysis buffer (Pierce RIPA Buffer, Cat. No. 89900; Thermo Fisher Scientific, Waltham, MA, USA) containing PMSF and the cocktail of protease inhibitors, and centrifuged at 13,000g for 15 min at 4ºC. The supernatants were supplemented with 5 µL of 5X reducing SDS buffer and boiled at 95°C for 10 min. Samples were separated at 130 V on a 15% gel (Rotiphorese Gel 30, Cat. No. 3029.2; Carl Roth, Karlsruhe, Germany) using Bio-Rad PowerPac Universal (Hercules, CA, USA). The gels were transferred to a PVDF membrane (size: 0.45 µm, Immobilon-P Transfer Membranes, Cat. No. IPVH00010; Merck Millipore, Burlington, MA, USA) for two hours at 80 V using the Bio-Rad Trans-Blot Turbo Transfer System (Hercules, CA, USA). After blotting, the membranes were washed with 1xTBS and blocked with 1% blocking reagent (Cat. No. 11921681001; Roche, Diagnostics GmbH, Mannheim, Germany) for 60 minutes. Membranes were then incubated overnight at 4°C with anti-SNX3 antibody (Cat. No. 10772-1-AP; Proteintech, Rosemont, IL, USA; 1:1000), diluted in 0.5% blocking buffer. After washing three times for 15 minutes with TBST (TBS with 0.05% Tween), the membranes were incubated for one hour at room temperature with anti-rabbit horseradish peroxidase (HRP)-conjugated secondary Ab (Jackson ImmunoResearch, West Grove, USA; 1:50,000), washed three times (10 minutes each) with TBST, and treated with the SignalFire (TM) Plus ECL reagent or SignalFire (TM) Elite ECL reagent (Cat. No. 12630S or 12757P respectively; Cell Signaling, Danvers, MA, USA). After recording the SNX3 signal, the membranes were washed three times for 15 minutes with TBST and incubated with mouse anti-IE1 mAb (clone CROMA101, 1mg/ml, 1:1) combined with mouse anti-actin Ab (Cat. No. MAB1501; Merck Millipore, Burlington, MA, USA; 1:80,000). The primary antibodies were resuspended in 0.5% blocking buffer and incubated overnight at 4ºC. Washing, incubation with the appropriate secondary antibody, and visualization were carried out as for SNX3.

Figure S3

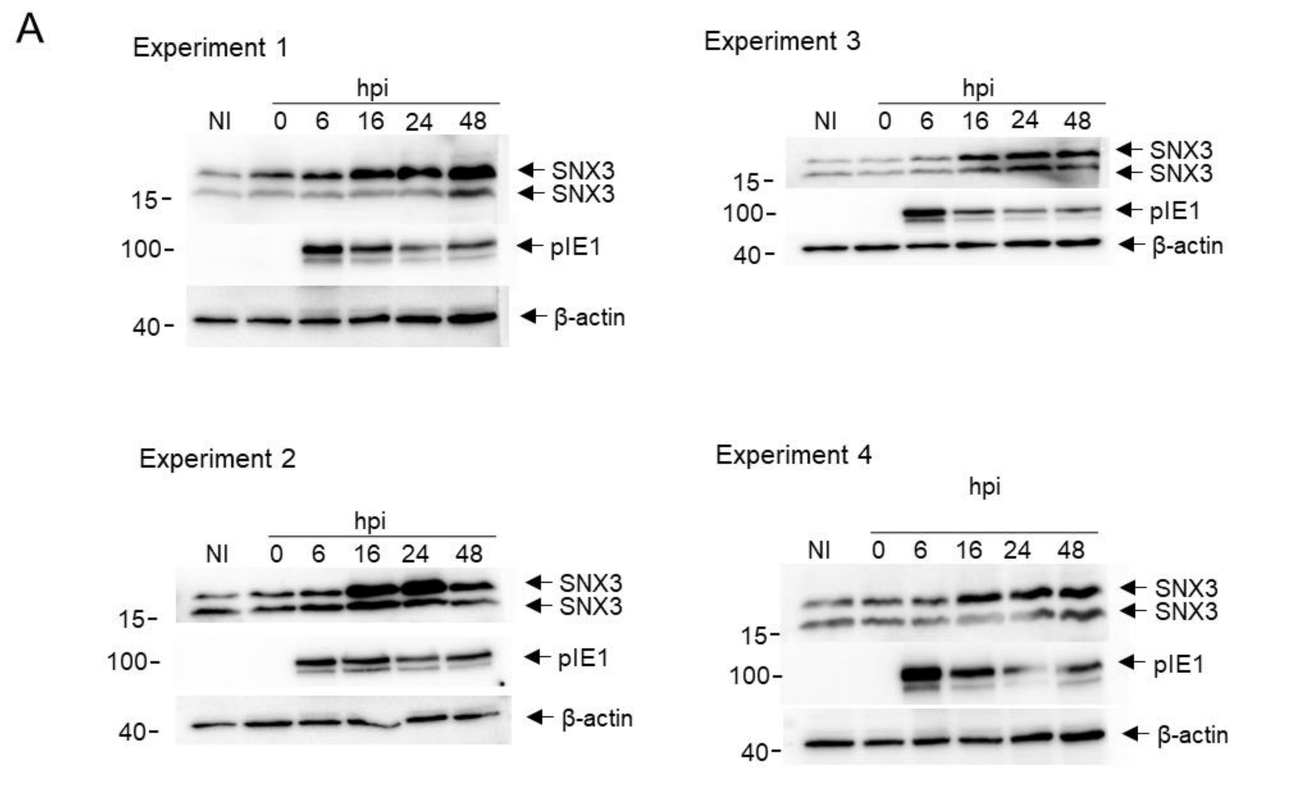
A
Experiment 1
hpi
NI 0 6 16 24 48
SNX3
SNX3
15-
100-
pIE1
40-
β-actin
Experiment 3
hpi
NI 0 6 16 24 48
SNX3
SNX3
15-
100-
pIE1
40-
β-actin
Experiment 2
hpi
NI 0 6 16 24 48
SNX3
SNX3
15-
100-
pIE1
40-
β-actin
Experiment 4
hpi
NI 0 6 16 24 48
SNX3
SNX3
15-
100-
pIE1
40-
β-actin

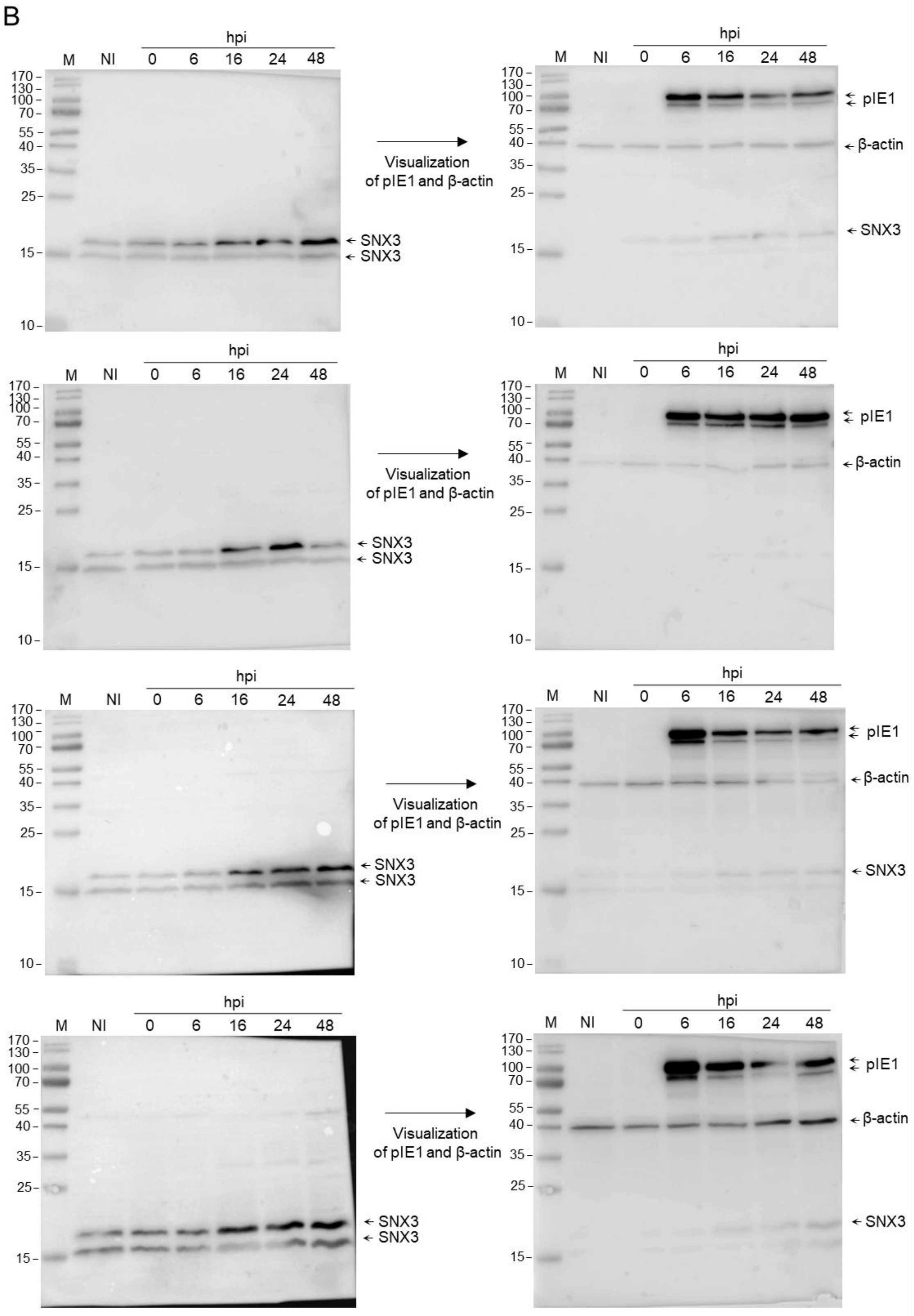

Figure S3. Western blot analysis of SNX3 expression during MCMV infection (*related to Figure 1D*). (**A**) Four independent experiments and (**B**) unprocessed Western blots of these experiments. NIH3T3 cells were infected with Δm138-MCMV (MOI 10) or left non-infected, lysed at 0, 6, 16, 24, 48 hpi, and analyzed for expression of SNX3. Expression of pIE1 and β-actin in each sample served as infection and loading controls, respectively, and was performed on the same membranes, as described in Figure S2.

Figure S4

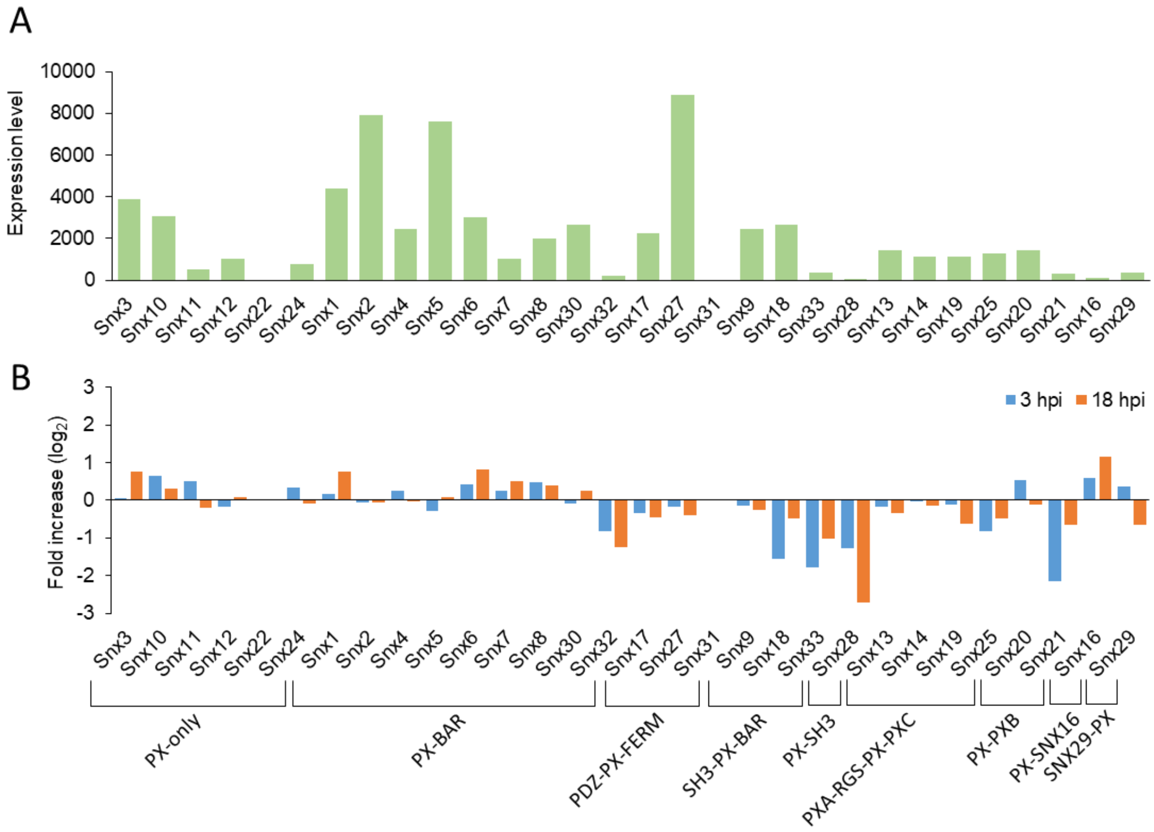

Figure S4. Effect of MCMV infection on the expression of the host cell genes encoding members of the SNX family (*related to Figure 1D*). (**A**) Absolute counts related to gene expression. (**B**) The data represent the fold change (log_2) of gene expression at the beginning (3 hpi) and the end (18 hpi) of the E phase of MCMV infection relative to the mock-infected cells. The data are extracted from our previously published transcriptome analysis (Lučin et. al, 2021).

Reference:

Lučin, P.; Jug Vučko, N.; Karleuša, L.; Mahmutefendić Lučin, H.; Blagojević Zagorac, G.; Lisnić, B.; Pavišić, V.; Marcelić, M.; Grabušić, K.; Brizić, I.; et al. Cytomegalovirus Generates Assembly Compartment in the Early Phase of Infection by Perturbation of Host-Cell Factors Recruitment at the Early Endosome/Endosomal Recycling Compartment/Trans-Golgi Interface. Front Cell Dev Biol 2020, 8, 914, doi:10.3389/fcell.2020.563607.

Figure S5

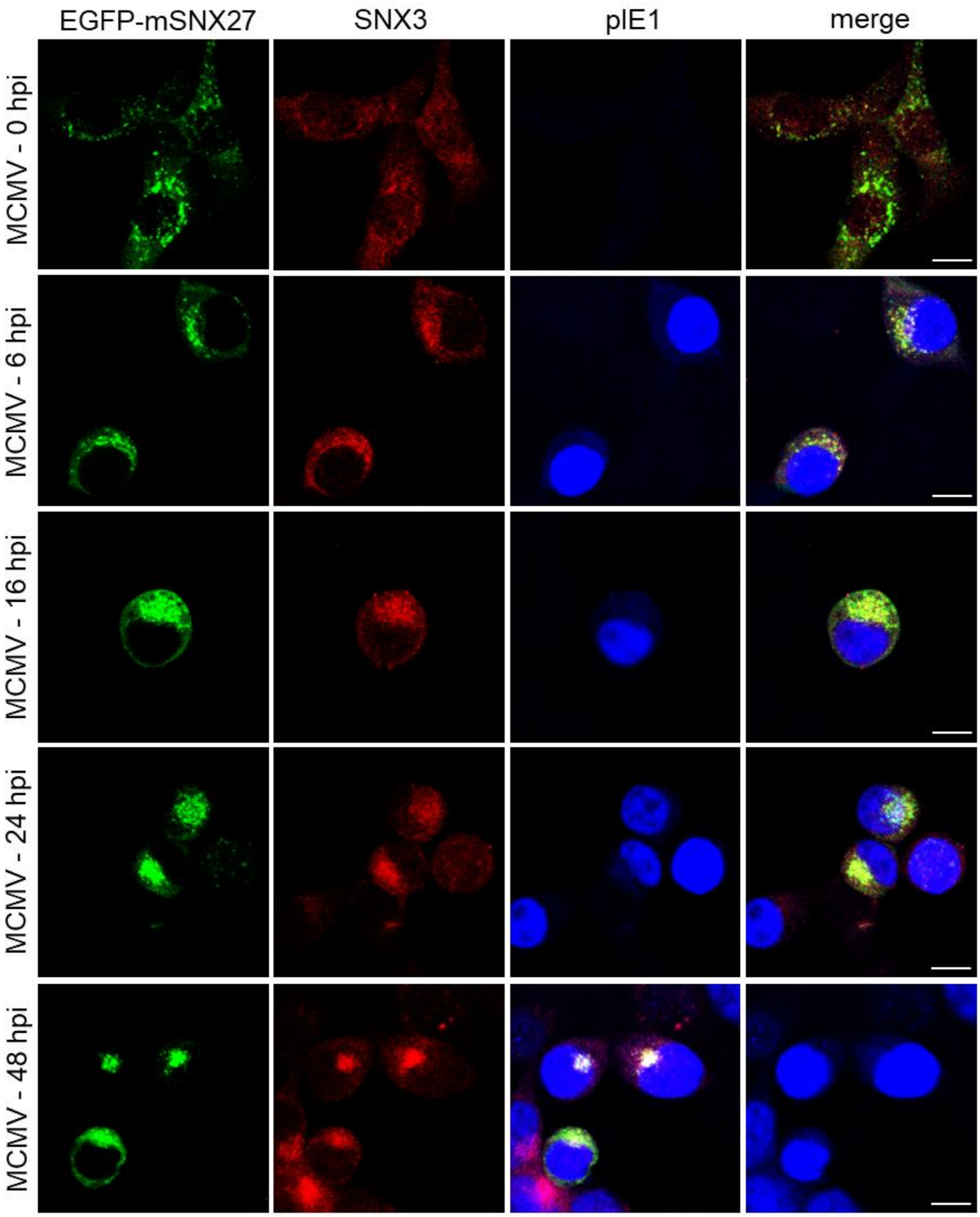

Figure S5. Colocalization analysis of SNX3 with SNX27. NIH3T3 cells with inducible expression of EGFP-mSNX27 were treated with doxycycline (2 μg/ml) for 24 hours and infected with Δm138-MCMV (MOI of 10). At 0, 6, 16, 24, and 48 hpi, cells were fixed, permeabilized, and stained with primary antibodies against SNX3 (red) and pIE1 (blue), followed by appropriate non-cross-reactive secondary antibodies, and analyzed by confocal microscopy. The representative confocal images are shown through the focal plane. Bars, 10 μm.

Figure S6

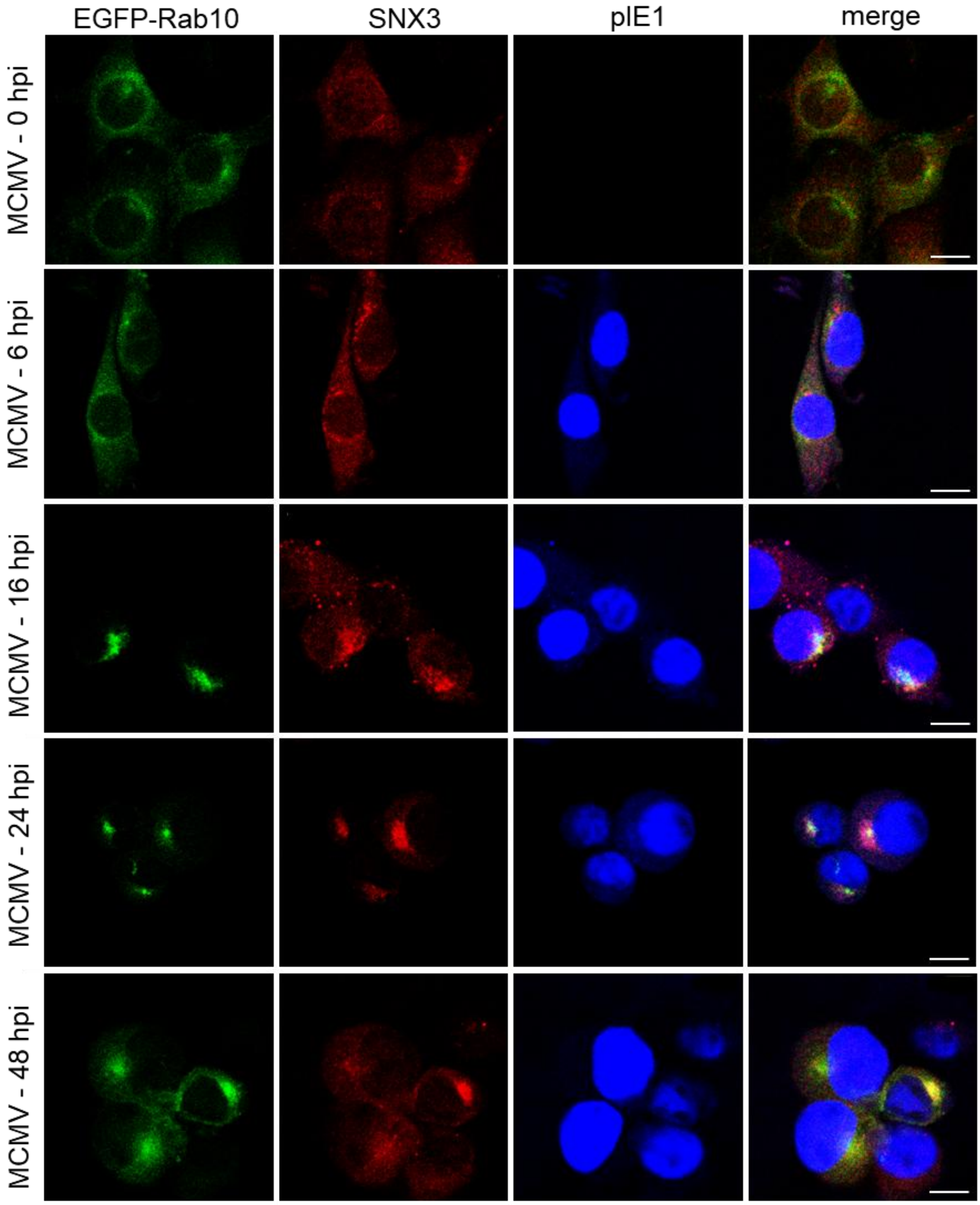

Figure S6. Colocalization analysis of SNX3 with Rab10. NIH3T3 cells with inducible expression of EGFP-Rab10 were treated with doxycycline (2 μg/ml) for 24 hours and infected with Δm138-MCMV (MOI of 10). At 0, 6, 16, 24, and 48 hpi, cells were fixed, permeabilized, and stained with primary antibodies against SNX3 (red) and pIE1 (blue), followed by appropriate non-cross-reactive secondary antibodies, and analyzed by confocal microscopy. The representative confocal images are shown through the focal plane. Bars, 10 μm.

Figure S7

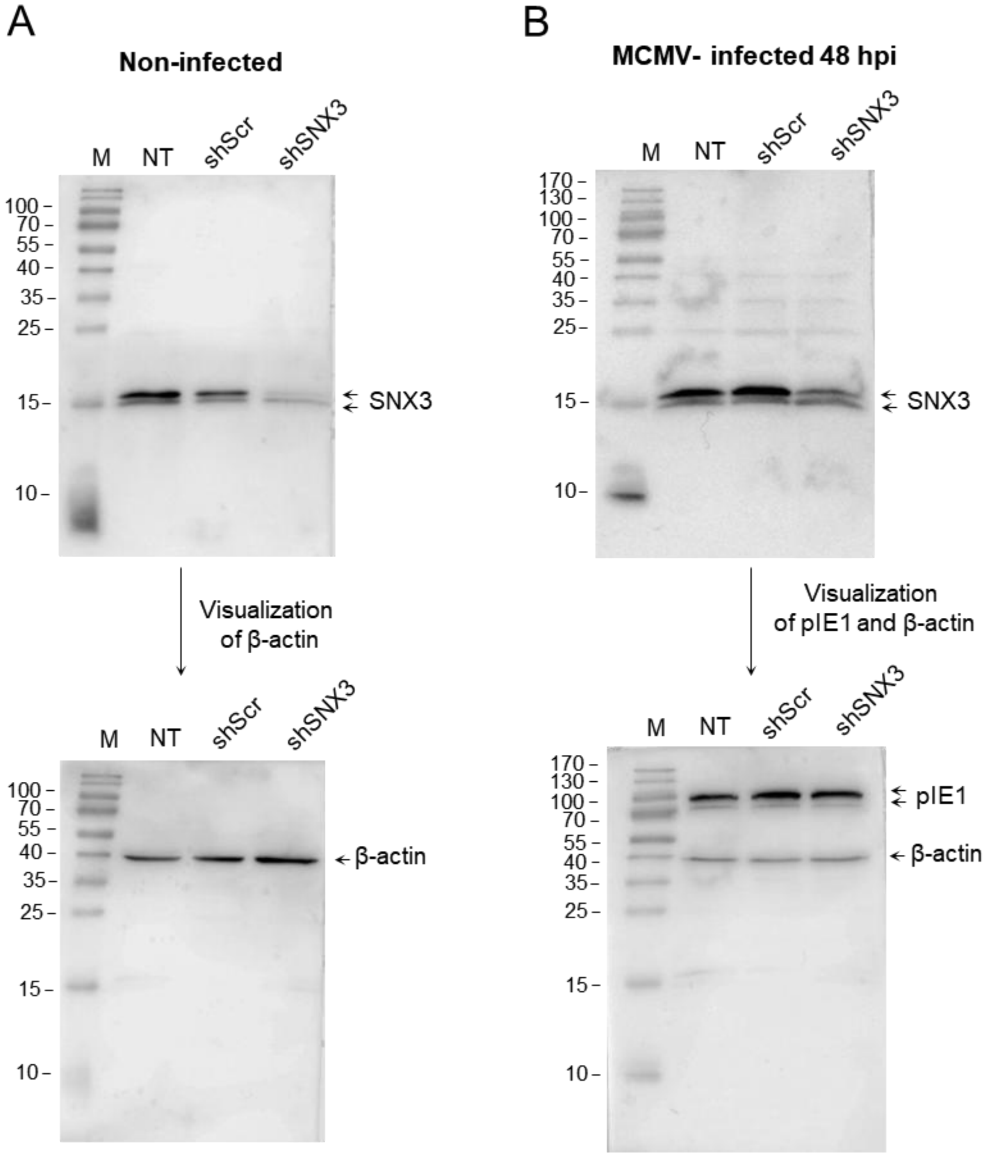

Figure S7. Unprocessed Western blots of SNX3 expression after SNX3 knockdown of non-infected and 48 hours MCMV-infected cells (*related to Figure 4C*). Overlaid original raw blots and unprocessed ECL images of SNX3, pIE1s, and β-actin expression in non-transfected (NT), and shScr- or shSNX3-expressing cells before (non-infected) (**A**) and 48 hours after infection (**B**) with Δm138-MCMV (MOI of 10), which were used as representative Western blots in Figure 4C of the manuscript. The western blotting membranes were first stained with Ab against SNX3. After visualization of SNX3 with secondary Ab, the membranes were washed and simultaneously stained with Abs against pIE1 and β-actin. The SNX3 protein is shown in the 18 kDa and 15 kDa forms. The molecular weight marker used in these experiments is PageRuler Prestained Protein Ladder (Thermo Fisher Scientific, Waltham, MA, SAD; Cat. No. 26616). M, marker line; NT, non-transfected.

NIH3T3 cells were plated in 12-well plates ($6x10^4$ cells/well) and infected the next day with Δm138-MCMV (virus 95.15) at an MOI of 10. Samples were collected before infection (non-infected) or 48 hours post infection. Non-transfected (NT) cells served as control. Cells were detached with a short trypsin/EDTA treatment (120 µl/well), trypsin was neutralized with 1.2 ml tissue culture medium containing 10% FCS, pelleted at 2,200 rpm for 2.5 min, and washed twice with PBS. The pellet was lysed on ice in 20 µL of lysis buffer (Pierce RIPA Buffer, Cat. No. 89900;

Thermo Fisher Scientific, Waltham, MA, USA) containing PMSF and the cocktail of protease inhibitors, and centrifuged at 13,000g for 15 min at 4°C. The supernatants were supplemented with 5 µL of 5X reducing SDS buffer and boiled at 95°C for 10 min. Samples were separated at 130 V on a 15% gel (Rotiphorese Gel 30, Cat. No. 3029.2; Carl Roth, Karlsruhe, Germany) using Bio-Rad PowerPac Universal (Hercules, CA, USA). The gels were transferred to a PVDF membrane (size: 0.45 µm, Immobilon-P Transfer Membranes, Cat. No. IPVH00010; Merck Millipore, Burlington, MA, USA) for two hours at 80 V using the Bio-Rad Trans-Blot Turbo Transfer System (Hercules, CA, USA). After blotting, the membranes were washed with 1xTBS and blocked with 1% blocking reagent (Cat. No. 11921681001; Roche, Diagnostics GmbH, Mannheim, Germany) for 60 minutes. Membranes were then incubated overnight at 4°C with anti-SNX3 antibody (Cat. No. 10772-1-AP; Proteintech, Rosemont, IL, USA; 1:1000), diluted in 0.5% blocking buffer. After washing three times for 15 minutes with TBST (TBS with 0.05% Tween), the membranes were incubated for one hour at room temperature with anti-rabbit horseradish peroxidase (HRP)-conjugated secondary Ab (Jackson ImmunoResearch, West Grove, USA; 1:50,000), washed three times (10 minutes each) with TBST, and treated with the SignalFire (TM) Plus ECL reagent or SignalFire (TM) Elite ECL reagent (Cat. No. 12630S or 12757P respectively; Cell Signaling, Danvers, MA, USA). After recording the SNX3 signal, the membranes were washed three times for 15 minutes with TBST and incubated with mouse anti-IE1 mAb (clone CROMA101, 1mg/ml, 1:1) combined with mouse anti-actin Ab (Cat. No. MAB1501; Merck Millipore, Burlington, MA, USA; 1:80,000). The primary antibodies were resuspended in 0.5% blocking buffer and incubated overnight at 4°C. Washing, incubation with the appropriate secondary antibody, and visualization were carried out as for SNX3.

Figure S8

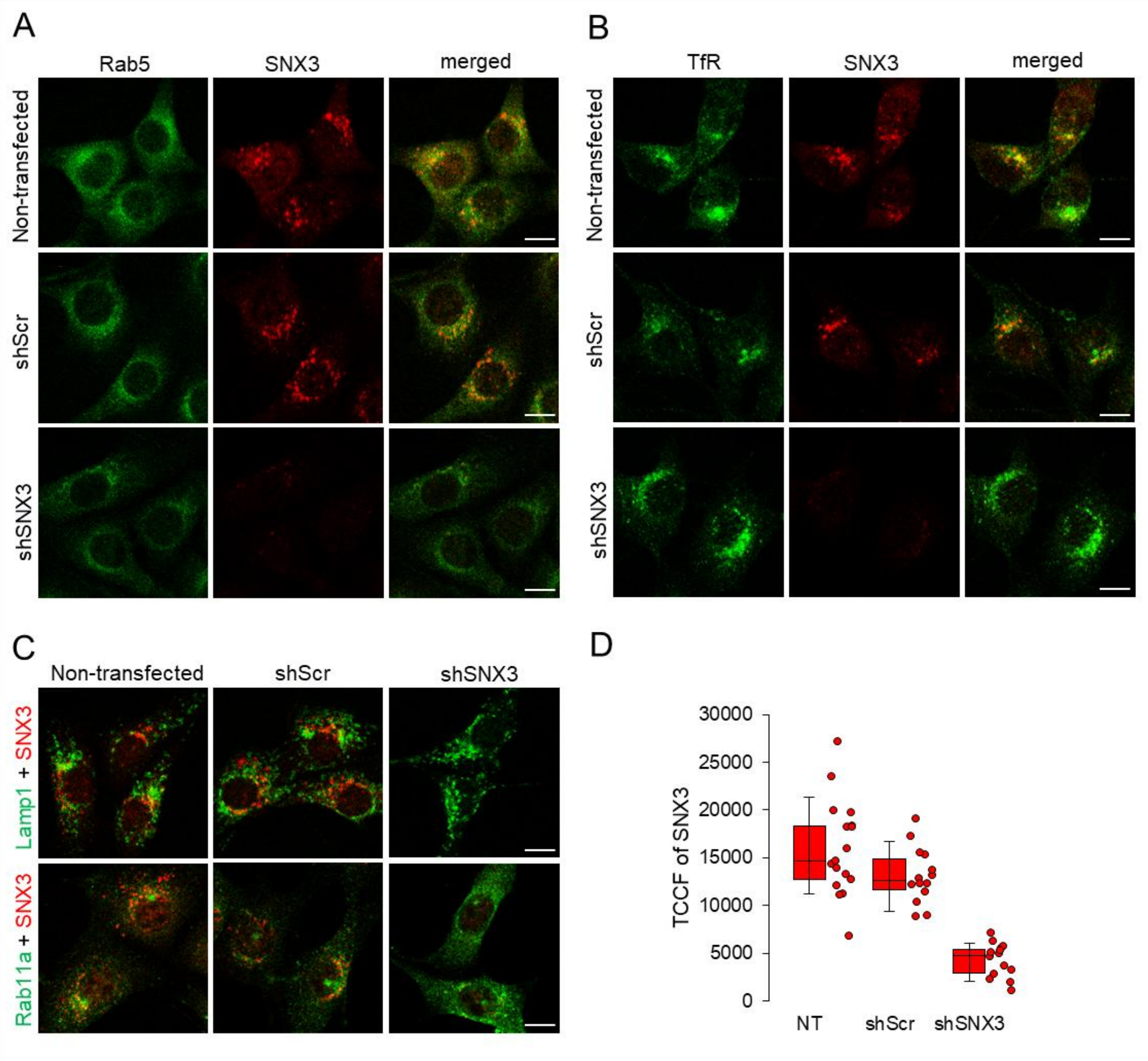

Figure S8. Expression of SNX3 shRNA has no effect on (**A**) early endosomes, (**B**) transferrin receptor trafficking, (**C**) late endosomes (Lamp1), and (**C**) endosomal recycling compartment (Rab11a). Immunofluorescence analysis of Rab5, Lamp1, and Rab11a in combination with SNX3 was performed on non-transfected (NT) NIH3T3 cells and cell lines expressing shScr and shSNX3. Transferrin receptor trafficking was observed after labelling with anti-TfR (clone R17 217.1.3) at 4°C for 30 minutes, followed by washout and 45 minutes of incubation at 37°C. Shown are confocal images through the focal plane of a representative experiment. Bars, 10 µm. (**D**) Total corrected cell fluorescence (TCCF) for SNX3 of each cell was quantified using ImageJ. Data show box-and-whisker plots and the result for each cell from two independent experiments.

Figure S9

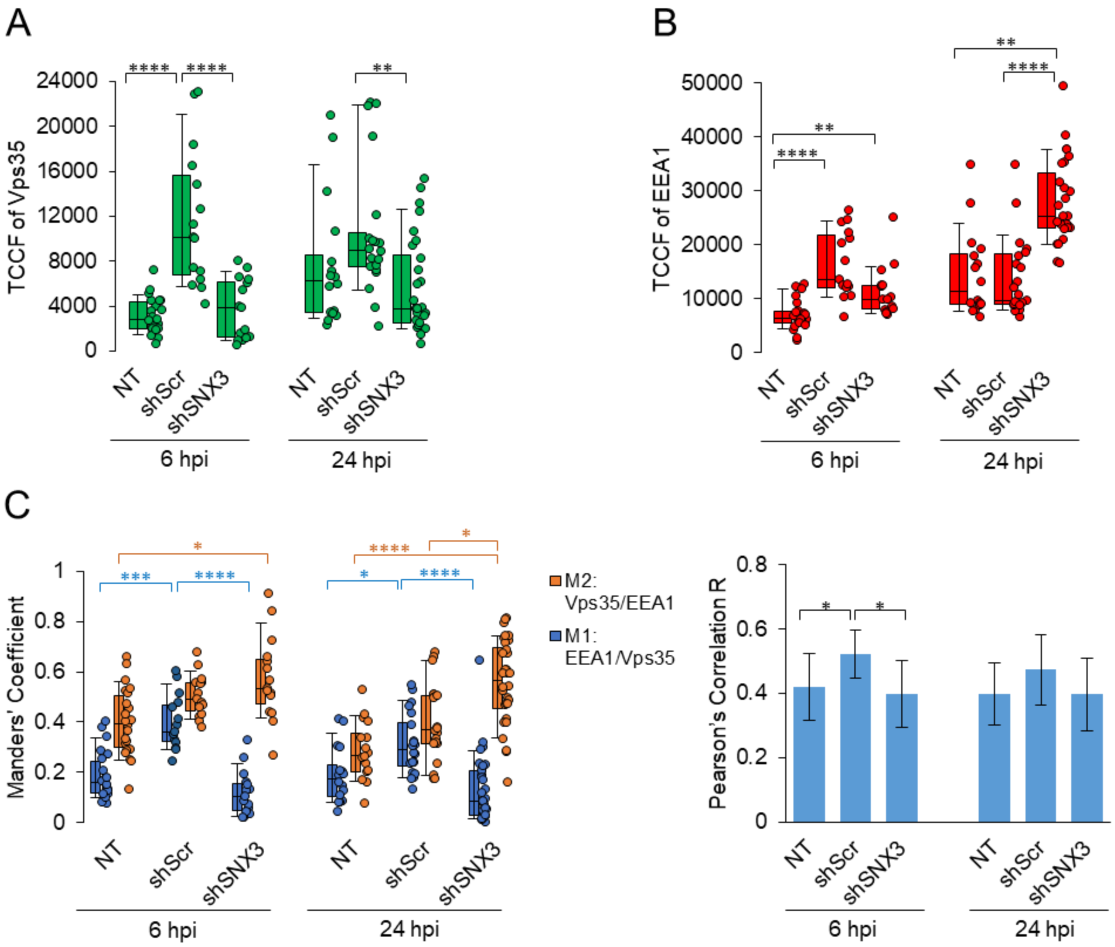

Figure S9. Membrane association of retromer, determined by Vps35, and early endosomal antigen 1 (EEA1) after the SNX3 knockdown (related to Figure 4)**.** Non-transfected (NT), shScr- or shSNX3-expressing NIH3T3 cells grown on coverslips were infected with Δm138-MCMV (MOI of 10). After 6 and 24 hpi, cells were fixed, permeabilized, and stained with antibodies against Vps35, EEA1, and pIE1. The staining was visualized with non-cross-reactive AF-conjugated secondary antibodies and analyzed by confocal imaging. Images were quantified (**A-B**) for total corrected cell fluorescence (TCCF) and (**C**) for 3D colocalization of Vps35 and EEA1. (**A-B**) TCCF of each cell was quantified with ImageJ. The data show box-and-whisker plots and the result for each cell from two independent experiments. (**C**) The 3D colocalization analysis of Vps35 with EEA1 is shown as the percentage overlap (Manders' coefficients) of EEA1 with Vps35 (M1) and Vps35 with EEA1 (M2), and as Pearson's correlation coefficient (R) per cell. The measurement was performed over the entire Z-stack with the Intermodes-algorithm threshold. The data in the left panel show box-and-whisker plots for M1/M2 and the result for each cell from two independent experiments, while the data in the right panel show the mean Pearson's correlation coefficient for these cells. The error bars show the standard deviation. Statistical significance was determined by the Kruskall-Wallis test with Dunn's multiple comparisons (*p < 0.05; ** p < 0.01; *** p < 0.001; **** p < 0.0001).

Figure S10

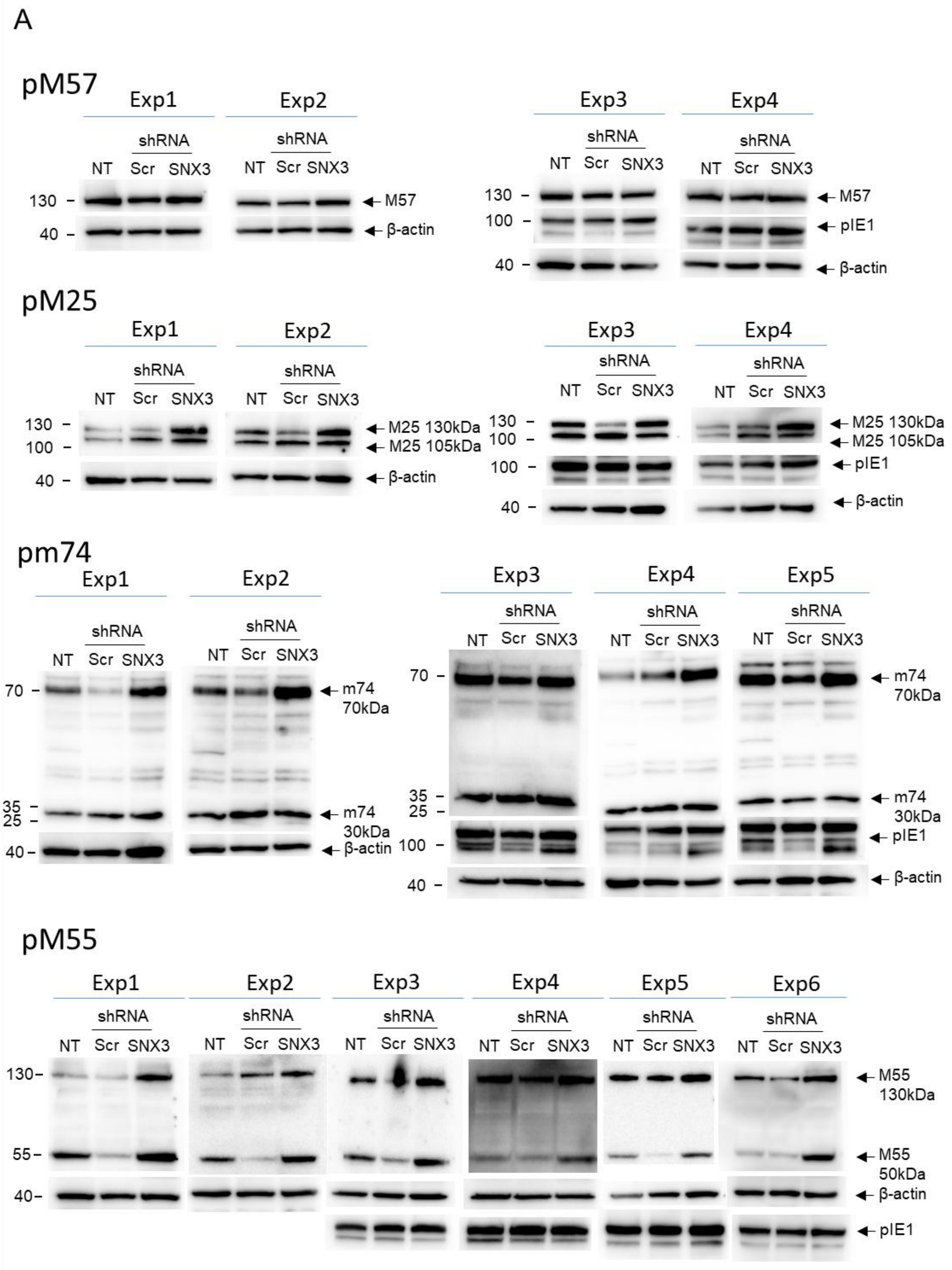
A
pM57
Exp1
Exp2
Exp3
Exp4
shRNA
NT Scr SNX3
130
40
100
M57
β-actin
pIE1
pM25
Exp1
Exp2
Exp3
Exp4
M25 130kDa
M25 105kDa
pm74
Exp5
m74 70kDa
m74 30kDa
70
35
25
pM55
Exp6
55
M55 130kDa
M55 50kDa

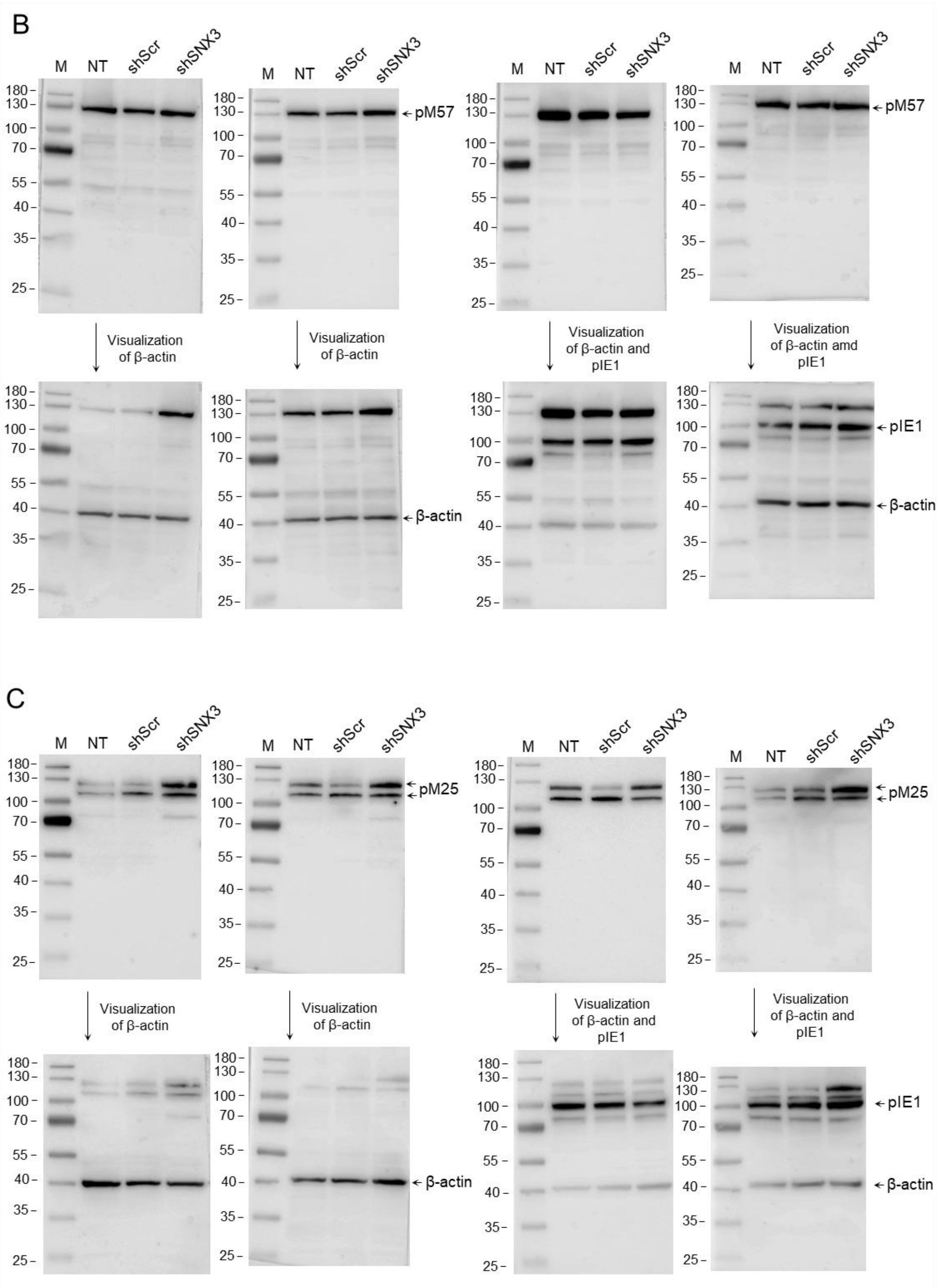
B
M
NT
shScr
shSNX3
180
130
100
70
55
40
35
25
pM57
Visualization of β-actin
Visualization of β-actin and pIE1
Visualization of β-actin amd pIE1
β-actin
pIE1
C
pM25

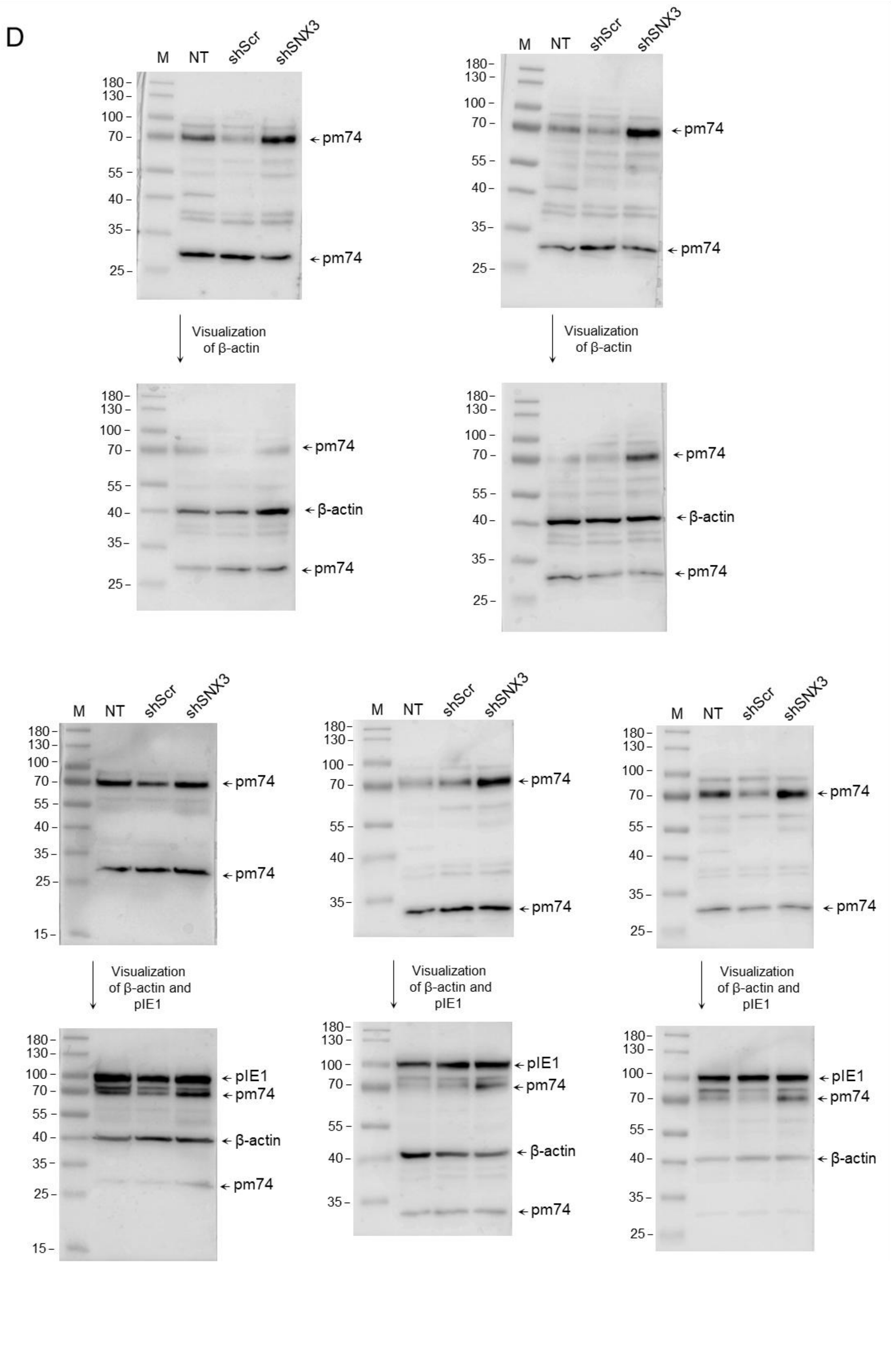
D
M
NT
shScr
shSNX3
180
130
100
70
55
40
35
25
15
pm74
β-actin
pIE1
Visualization of β-actin
Visualization of β-actin and pIE1

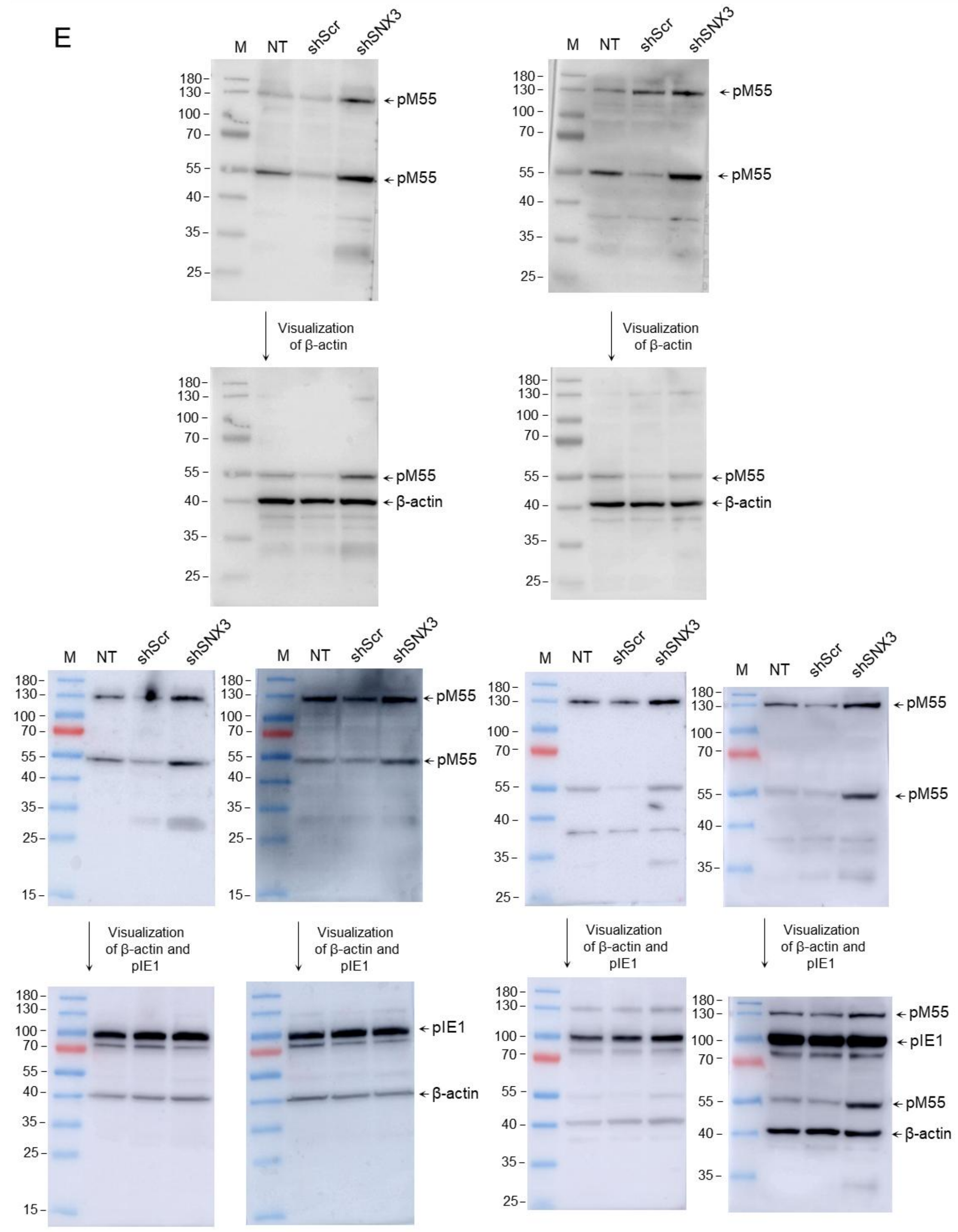

Figure S10. Western blot analysis of MCMV protein expression in shScr- and shSNX3-expressing cells (*related to Figure 4G*). (**A**) Summary of independent experiments and (**B-E**) unprocessed Western blots of these experiments. NIH3T3 cell lines expressing shScr, shSNX3, and non-transfected (NT) NIH3T3 cells were infected with Δm138-MCMV (MOI 10) and analyzed by Western blot 48 hpi. The expression of the viral proteins pM57, pM25 (130- and 105-kDa form), pM55 (130- and 55-kDa form), and pm74 (70- and 30-kDa form) was determined in 4-6 independent Western blots (Exp1-Exp6). Loading control (β-actin) and pIE1 expression were determined on the same membrane as described in Figure S2. Band density was quantified using ImageQuantTL software (version 10.2). Results are normalized to β-actin expression in the same sample. For presentation in Figure 4G, the signal level of shScr- and shSNX3-expressing cells was normalized to the non-transfected control.

Figure S11

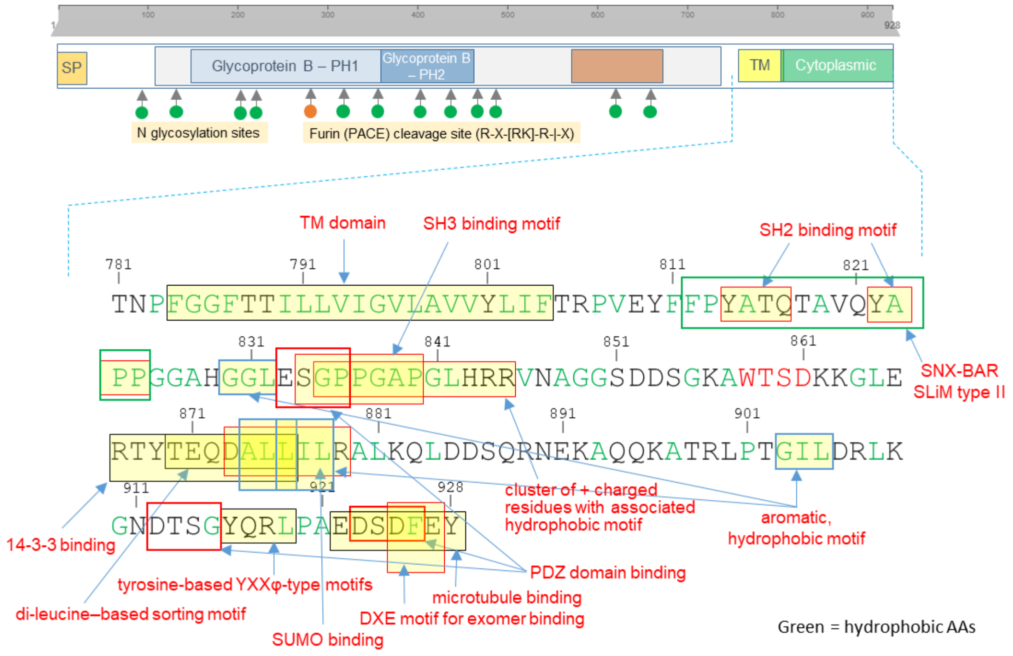

Process	Ligand	Consensus motif	MCMV gB motifs	Trafficking route
Clathrin-dependent processes	AP1	*Ex*xLL	TEQDALL	TGN-to-EE, EE-to-TGN
		YXXL	YQRL	Sorting to the TGN
	AP2	*Ex*xLL	TEQDALL	PM-to-EE
		YXXL	YQRL	
	AP3	*Ex*xLL	TEQDALL	TGN-to-LE
	PACS1 and PACS2	Acidic cluster	Not present	
	ACAP1 (BAR)	cluster of + charged residues together with associated hydrophobic motif	GPPGAPGLHRR	RE/ERC-to-TGN<br>RE/ERC-to-PM
Retreiver binding	SNX17	ΦxNPx[F/Y]; ΦxNxx[F/Y]	Not present	EE/TRE-to-PM
	COMMD1	NPxY	Not present	EE/TRE-to-PM
Retromer binding	SNX27 (PDZ domian)	$[E/D]^{-3}[S/T]^{-2}\ x\ ^{-1}\Phi^{0}$	DSDF	EE/TRE-to-PM
	SNX27 (FERM-like domain)	ΦxNPxpY; ΦxNxxpY	Not present	EE/TRE-to-PM
	SNX3:VPS26:VPS35:VPS29	Φx[L/M/V]<br>(aromatic hydrophobic motif)	GGL, ALL, GIL, LIL	EE-to-TGN
	VPS26:VPS35:VPS29	FANSHY	Not present	
SNX-BAR binding	SNX1/SNX2:SNX5/SNX6	$[FYW]X[FY]X_{3\text{-}15}\Phi X\Phi$	FPYATQTAVQYAPP	EE-to-PM
ARF GTPase binding	GGA1	DIpSLL	Not present	TGN-to-EE
	GGA3	DxxL[L/V]	Not present	TGN-to-EE

TGN, trans-Golgi network; EE, early endosome; PM, plasma membrane; ERC, endosomal recycling compartment; TRE, tubular recycling endosome;

Figure S11. Analysis of short linear motifs (SLiMs) in the cytoplasmic domain of MCMV glycoprotein B (gB) that can be related to EE sorting processes (Cullen and Steinberg, 2018; Kumar et al., 2024). Protein sequence was extracted from UniProt (https://www.uniprot.org/). Transmembrane domain was found using DeepTMHMM (Hallgreen et al., 2022).

References:

Cullen, P.J.; Steinberg, F. To Degrade or Not to Degrade: Mechanisms and Significance of Endocytic Recycling. Nat Rev Mol Cell Biol 2018, 19, 679–696, https://doi.org/10.1038/s41580-018-0053-7.

Kumar et al., ELM—the Eukaryotic Linear Motif resource—2024 update. Nucleic Acids Research, Volume 52, Issue D1, 5 January 2024, Pages D442–D455, https://doi.org/10.1093/nar/gkad1058.

Hallgren, J.; Tsirigos, K.D.; Pedersen, M.D.; Armenteros, J.J.A.; Marcatili, P.; Nielsen, H.; Krogh, A.; Ole Winther, O. (). DeepTMHMM predicts alpha and beta transmembrane proteins using deep neural networks. BioRxiv 2022, https://doi.org/10.1101/2022.04.08.487609.
